# Evaluation of the PTW microDiamond in edge‐on orientation for dosimetry in small fields

**DOI:** 10.1002/acm2.12906

**Published:** 2020-05-22

**Authors:** Owen J. Brace, Sultan F. Alhujaili, Jason R. Paino, Duncan J. Butler, Dean Wilkinson, Brad M. Oborn, Anatoly B. Rosenfeld, Michael L. F. Lerch, Marco Petasecca, Jeremy A. Davis

**Affiliations:** ^1^ Centre for Medical Radiation Physics University of Wollongong Wollongong NSW Australia; ^2^ Australian Radiation Protection and Nuclear Safety Agency (ARPANSA) Yallambie VIC UK; ^3^ Illawarra Cancer Care Centre Wollongong Hospital Wollongong Wollongong NSW Australia

**Keywords:** angular dependence, IAEA TRS‐483 CoP, PTW microDiamond, small field dosimetry

## Abstract

**Purpose:**

The PTW microDiamond has an enhanced spatial resolution when operated in an edge‐on orientation but is not typically utilized in this orientation due to the specifications of the IAEA TRS‐483 code of practice for small field dosimetry. In this work the suitability of an edge‐on orientation and advantages over the recommended face‐on orientation will be presented.

**Methods:**

The PTW microDiamond in both orientations was compared on a Varian TrueBeam linac for: machine output factor (OF), percentage depth dose (PDD), and beam profile measurements from 10 × 10 cm^2^ to a 0.5 × 0.5 cm^2^ field size for 6X and 6FFF beam energies in a water tank. A quantification of the stem effect was performed in edge‐on orientation along with tissue to phantom ratio (TPR) measurements. An extensive angular dependence study for the two orientations was also undertaken within two custom PMMA plastic cylindrical phantoms.

**Results:**

The OF of the PTW microDiamond in both orientations agrees within 1% down to the 2 × 2 cm^2^ field size. The edge‐on orientation overresponds in the build‐up region but provides improved penumbra and has a maximum observed stem effect of 1%. In the edge‐on orientation there is an angular independent response with a maximum of 2% variation down to a 2 × 2 cm^2^ field. The PTW microDiamond in edge‐on orientation for TPR measurements agreed to the CC01 ionization chamber within 1% for all field sizes.

**Conclusions:**

The microDiamond was shown to be suitable for small field dosimetry when operated in edge‐on orientation. When edge‐on, a significantly reduced angular dependence is observed with no significant stem effect, making it a more versatile QA instrument for rotational delivery techniques.

## 1 INTRODUCTION

Quality assurance (QA) for small field radiotherapy is a challenging task requiring new detectors and QA methodologies. Codes of Practice (CoP) for conventional external photon beam radiotherapy are not suitable for small field dosimetry as they do not account for the lack of lateral charged particle equilibrium (LCPE) or occlusion effects.^1^

The IAEA TRS‐483 CoP for small field dosimetry recommends detectors used for QA should be: small relative to the minimum field size and the range of the secondary charged particles, have a high signal to noise ratio (SNR), high spatial resolution and also be energy, dose rate, and angular independent in response. Volume averaging effects can be avoided by the use of detectors with submillimeter spatial resolution, such as the PTW microDiamond,^2^, ^3^, ^4^, ^5^ IBA razor diode detector,^6^ and the edgeless silicon diode,^7^ however, these detectors do not address some of the more serious perturbation effects. A tissue equivalent, small volume detector can be made for plastic scintillators but these detectors suffer from a large temperature and humidity dependence as well as nonlinearity at low doses.^8^ In other detectors, density‐based perturbation effects are caused by the inhomogeneity of the detector volume and packaging with respect to the surrounding medium. Perturbation is created due to the mismatch in stopping power ratios of the detector and its packaging materials relative to water which can lead to large variations in the detector response. Alfonso et al, presented a methodology where the detector response variation with field size can be corrected for by using a detector‐specific sensitivity correction factor, however, this assumes a certain detector orientation, angular independent response, and isocentric delivery.^9^

Diamond is a natural candidate for small field dosimetric applications given its tissue equivalence,^10^, ^11^, ^12^ radiation hardness,^13^, ^14^ and near energy independent response to water for x rays.^15^ The uptake of diamond‐based devices has been hampered by its lower sensitivity which can be quantified in the energy required to create an electron hole pair (E_e/h_ = 13 eV^16^). Furthermore, the density of diamond (*ρ* = 3.52 g cm^−3^) will increase perturbation effects for small field dosimetry QA. The PTW microDiamond (PTW 60019, PTW, Freiburg, Germany) is currently one of the most prolific diamond‐based detector in clinical use in radiotherapy. The microDiamond detector developed by the University of Rome Tor Vergata^17^, ^18^ and commercialized by PTW, utilizes synthetic single crystal diamond featuring a metal/intrinsic diamond/p‐type diamond (m‐i‐p+) structure. The result of the m‐i‐p + structure is a built‐in potential allowing for the device to run in passive mode, that is, zero applied bias. There exists within the literature an ongoing debate regarding the appropriateness of the PTW microDiamond for small field dosimetry with conflicting reports of over response,^2^, ^3^ water equivalence,^19^, ^20^, ^21^, ^22^ and under response for fields <1 cm^2^. Recent work has also quantified an additional effect of radiation‐induced charge imbalance, in the PTW microDiamond.^23^ At field sizes of length <2 cm radiation induced charge in the electrical contact of the PTW microDiamond is reported to result in an overresponse of the device. Despite these limitations for dosimetry applications the microDiamond remains an area of interest for QA with its response having recently been characterized for use in an MRI linac^24^ and for the MRI‐associated surface dose from electron contamination.^25^

One of the key advantages of the PTW microDiamond is its high spatial resolution when operating in edge‐on orientation,^21^, ^26^, ^27^ however, the IAEA TRS‐483 CoP requirement of a face‐on orientation for all QA measurements means that this micron scale spatial resolution is unrealized. The edge‐on and face‐on orientation are referred to by the terms perpendicular and parallel orientation in the CoP, respectively. This edge‐on (perpendicular) orientation has not previously been characterized in the context of small field dosimetry. Furthermore, diamond detectors have been investigated for both stereotactic ablative radiotherapy (SABR) and volumetric arc therapy (VMAT),^28^, ^29^ making the angular dependence of this orientation of great interest. In this study, the PTW microDiamond is characterized in both edge‐on and face‐on orientations for the first time. Additionally, the angular dependence of the PTW microDiamond is investigated thoroughly in order to evaluate the potential use in edge‐on orientation.

## 2 MATERIALS AND METHODS

**Fig. 1:**
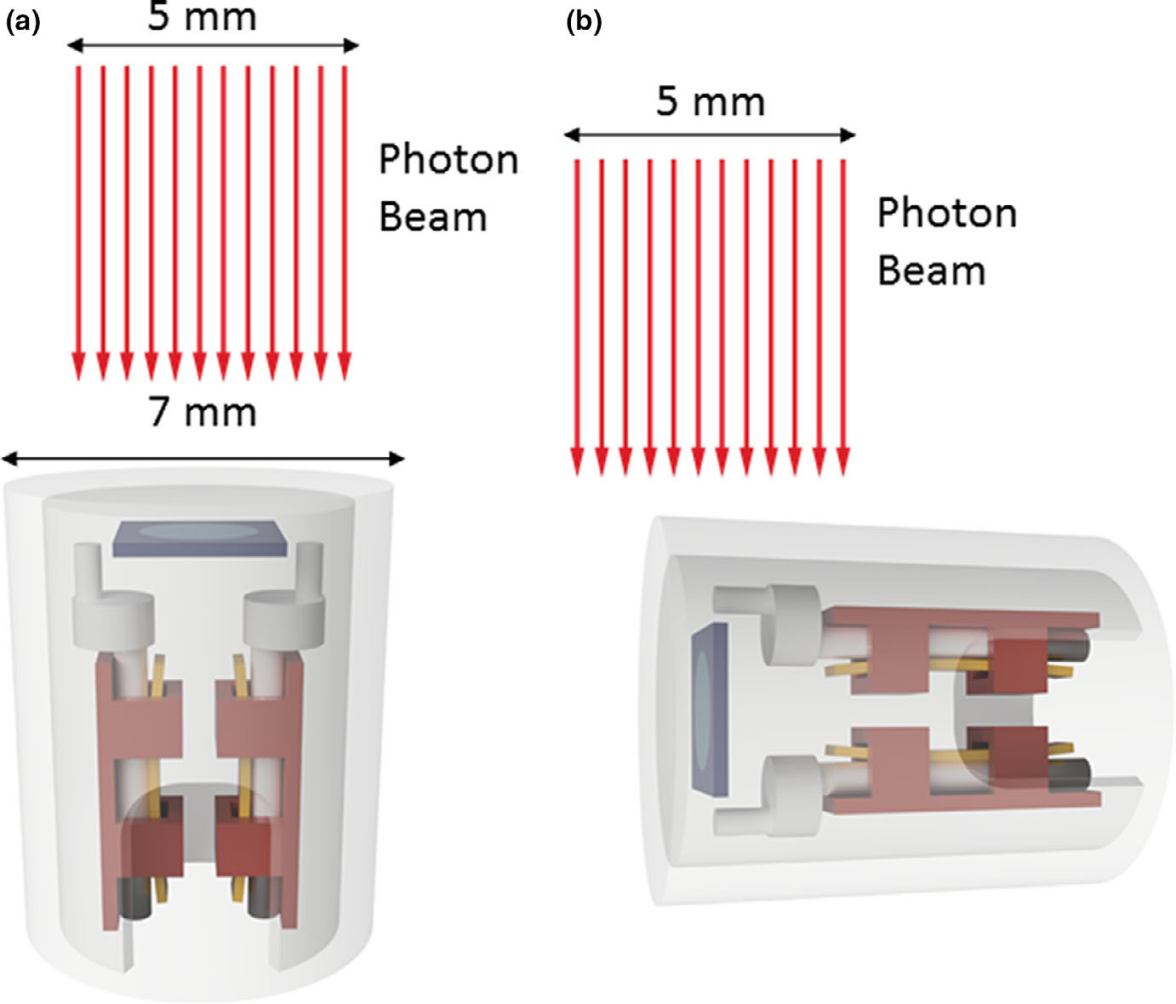
(a) Face‐on orientation for PTW microDiamond with an incident photon beam of field edge size 5 mm (b) edge‐on orientation. The microDiamond has a centered rectangular representing the diamond size with central disc (blue) representing, to scale, the 1 µm thick sensitive volume

Two orientations of the PTW microDiamond detector are investigated in this study, face‐on and edge‐on as depicted in Fig. 1. The face‐on orientation is the standard practice recommended by the manufacturer with a requirement of the IAEA TRS‐483 CoP. As demonstrated in Fig. 1(a) with the microDiamond in a face‐on orientation there is a 2.2‐mm diameter sensitive volume^30^; for small fields this could results in a volume averaging effect. To better utilize the detector's 1‐µm thick sensitive volume, this work characterizes the response of the detector when positioned in edge‐on orientation with respect to the incident photon beam. The PTW microDiamond orientation comparison was assessed primarily on a TrueBeam linac (Varian Medical Systems, Palo Alto, CA). Both 6‐MV flattening filter (6X) and flattening filter‐free (6FFF) modalities were investigated. To that end, the PTW microDiamond was used to determine percentage depth dose (PDD) curves, beam profiles, and output factors (OF) within a 3D water tank (Blue Phantom 2, IBA Dosimetry, Schwarzenbruck, Germany) in face‐on and edge‐on orientation.

### 2.A Percentage depth dose measurements

In order to assess the appropriateness of the microDiamond for small field the PDD measurements were performed for 1 × 1 and 3 × 3 cm^2 ^fields. Measurements were started from the maximum depth of 31 cm and moved up to the surface to avoid any error in depth from surface tension. The detectors were aligned to the surface of the water at their effective point of measurement. The one exception to this alignment method is the microDiamond in edge‐on orientation as the effective point of measurement has not been previously reported and was aligned at the center of the sensitive volume. A source to surface distance (SSD) of 100 cm was used. Additional detectors used for comparison were the IBA Razor diode and IBA Razor ionization chamber (IBA Dosimetry, Schwarzenbruck). A list of detectors used in this study and their corresponding sensitive volumes is presented in Table 1.

**Table 1:** List of detectors used, their sensitive volumes, and corresponding dimensions (note all volumes approximately cylindrical)

Detector	Sensitive volume (mm^3^)	Diameter (mm)	Length (mm)
IBA RAZOR DIODE	0.006	0.6	0.02
IBA razor chamber	10	2.0	3.6
PTW microDiamond	0.004	2.2	0.001
CC01	10	2.0	3.6

### 2.B Lateral beam profiles

To assess the impact of orientation on the spatial resolution of the microDiamond for small field QA, field profiles were performed for 0.5 × 0.5, 1 × 1, and 3 × 3 cm^2^ fields in face‐on and edge‐on orientations at 10 cm depth. The profiles were performed at 100 cm SSD with jaw defined field for in‐plane and cross‐plane profiles, with multiple measurements taken at each point and averaged. The field sizes are defined for the beam at an SSD of 100 cm.

### 2.C Output factor

Output factors for MLC defined fields were measured with the microDiamond for field sizes between 0.5 × 0.5 and 8 × 8 cm^2^ and normalized against response within 10 × 10 cm^2^. The detectors were positioned 10 cm deep with an SSD of 90 cm. The detector alignment with respect to the center of the field was validated by performing field profiles of the 0.5 × 0.5 cm^2^ MLC defined field. Complementary measurements were performed with the IBA Razor diode, IBA Razor ionization chamber, and CC01 ionization chamber for comparison.

### 2.D Angular dependence

**Fig. 2:**
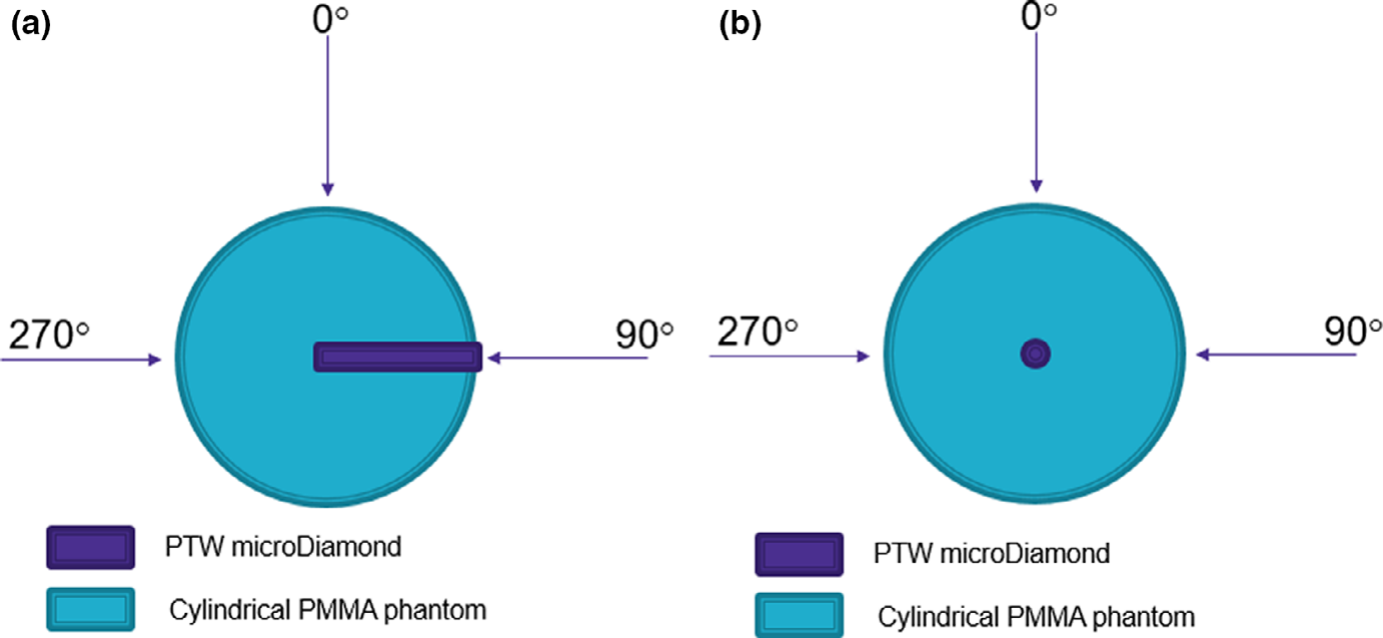
Experimental set‐up for angular dependence measurement. (a) microDiamond orientation to beam direction in face‐on phantom (b) microDiamond orientation to beam direction in edge‐on phantom

Angular dependence measurements for face‐on and edge‐on orientation were performed using a Clinac IX (Varian Medical Systems, Palo Alto, CA). The experimental set‐up is depicted in Fig. 2 which shows two cylindrical phantoms identical in size (diameter 30 cm and thickness 10 cm) but different only in the position of the detector insert. The first phantom (Fig. 2(a)) features a detector insert that is through the radial side of the phantom which was used to investigate the angular dependence of the PTW microDiamond, as a function of field size, in face‐on orientation. The second phantom [Fig. 2(b)] features a detector insert that is through the flat face of the phantom that was used to investigate the angular dependence of the PTW microDiamond, in the edge‐on orientation. The depth of the inserts was such that the center of the sensitive volume (SV) of the PTW microDiamond would be at isocenter, regardless of gantry rotation. All measurements are with a 6X photon beam, dose rate of 600 MU/min, and delivery of 100 MU repeated three times. Field sizes between 0.5 × 0.5 cm^2^ to 10 × 10 cm^2^ were used at every 15° increment within the region 120° either side of the 0° gantry position. Prior to the measurements, the detector is aligned to the center of the 0.5 × 0.5 cm^2^ field using two motorized stepping stages.

### 2.E Stem effect

**Fig. 3:**
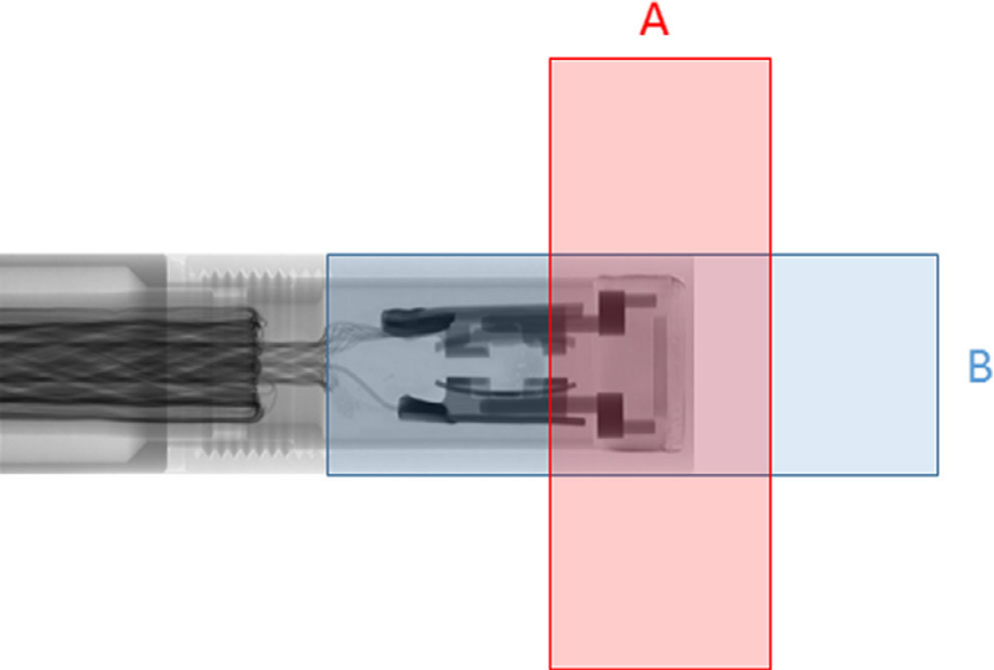
Orientations of rectangular fields **A** perpendicular and **B** parallel to the stem of the microDiamond to evaluate the stem effect

The IAEA TRS‐483 CoP does not recommend using devices in an edge‐on mode for profile scanning because of the potential for the introduction of extra cameral current in the detector stem or cable, but if used, this effect should be corrected for. The stem effect was quantified for the microDiamond in edge‐on orientation for both 6X and 6FFF. Rectangular fields of size 1 × 10, 3 × 10, and 1 × 3 cm^2^ were used to measure the impact on the microDiamond output factor. The fields were shaped with MLCs and centered on the sensitive volume of the microDiamond using stepper motor stages. A measurement was taken with the long field edge lined perpendicular to the cable, Fig.. 3 (**A**) and then the collimator was rotated 90° to have the long edge parallel to the cable, Fig. 3 (**B**). The percentage difference in output factor was taken as a measure of the stem effect ((**B **− **A**)/**A** × 100). The microDiamond was inserted at the center of 30 × 30 cm^2^ SW blocks at 10 cm depth with an SSD of 100 cm.

### 2.F TPR_20,10_

Tissue to Phantom Ratios (TPR) were obtained for the microDiamond in edge‐on mode as well as the IBA Razor diode and compared with the CC01 ionization Chamber. In this work the TPR_20,10_ (*S*) is defined as the ratio of detector response *R* at 20 cm depth to 10 cm, R_20_/R_10_. *S* denotes square field size ranging between 0.5 × 0.5 cm^2^ and 10 × 10 cm^2^ *.* A source to detector distance (SDD) of 100 cm was used with 30 × 30 cm^2^ blocks of SW which was used to make the relevant build‐up depth and 10 cm of back scatter material.

## 3 RESULTS

### 3.A Percentage depth dose measurements

**Fig. 4:**
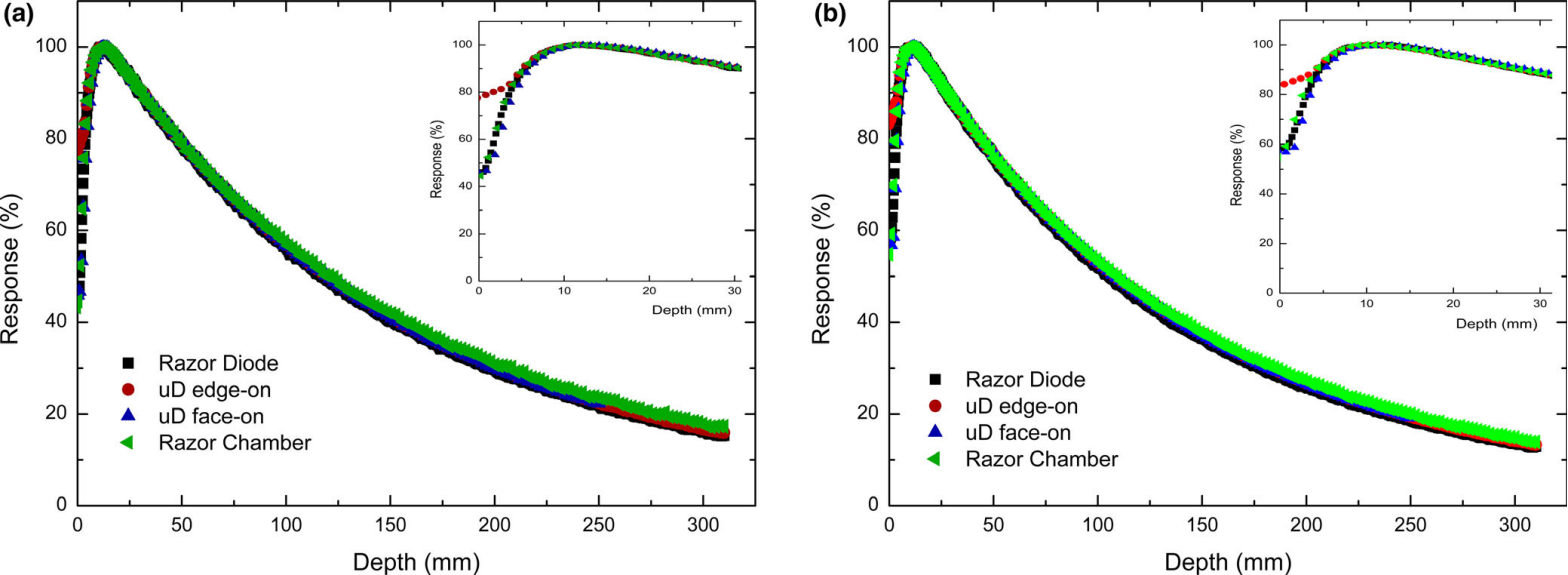
Percentage depth dose measurements of 1 × 1 cm^2^ jaw defined field performed in IBA blue water two phantom on Varian TrueBeam with IBA Razor diode (black square), PTW microDiamond in edge‐on (red circle) and face‐on (blue diamond) orientations for 6X (a) and 6FFF (b)

**Fig. 5:**
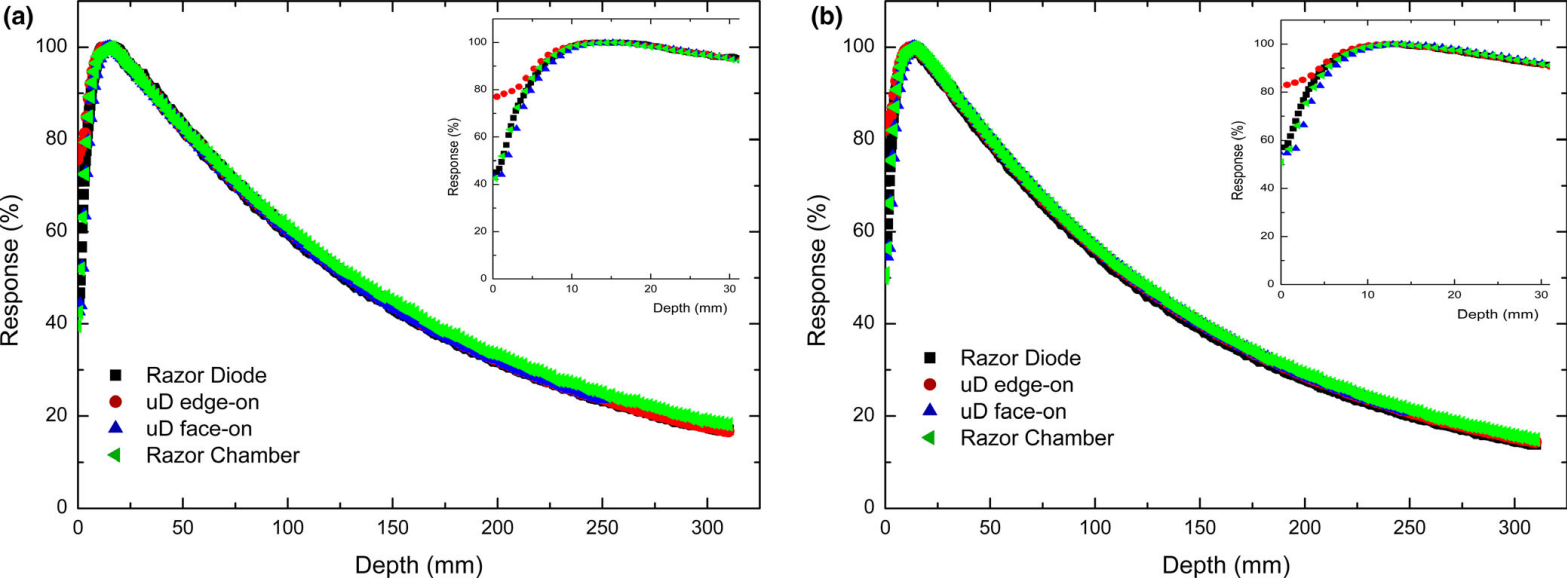
Percentage depth dose measurements of 3x3cm^2^ jaw defined field performed in IBA blue water two phantom on Varian TrueBeam with IBA Razor diode (black square), PTW microDiamond in edge‐on (red circle) and face‐on (blue diamond) orientations for 6X (a) and 6FFF (b)

Figures 4 and 5 show the PDD curves for a 1 × 1 and 3 × 3 cm^2^ field respectively, and demonstrate good agreement between all four data sets. The only notable difference in the PDD curves is the overresponse of the PTW microDiamond (edge‐on) in the build‐up region which is depicted in the zoomed in region of Figs. 4 and 5. This discrepancy is attributed to the edge‐on orientation of the microDiamond at depths shallower than 3.5 mm having part of the detector above the surface of the water. The microDiamond in face‐on orientation agrees closely with the response of the IBA razor diode and chamber in the build‐up region indicating that the diamond and associated packaging do not produce any significant perturbation effect in this region. It is worth noting that both orientations are influenced by volume averaging effects. In edge‐on the detector will average over a greater range of depths while in face‐on the measurement averages off axis components of the beam which becomes less critical as the depth increases and the field diverges. This does not appear to be significant as the microDiamond (edge‐ on) comes into agreement with the response of the Razor chamber after 5 and 10 mm depth for the 1 × 1 and 3 × 3 cm^2^ fields respectively indicating its appropriateness for PDD measurements as typical commissioning with QA measurements mainly concerned with depths greater than or equal to d_max_.

The depth of d_max_ for a 1 × 1 cm^2 ^and a 3 × 3 cm^2^ square field are reported in Table 2. The values of d_max _for the four data sets show variability of depths measured of up to 2.2 mm. The microDiamond in edge‐on orientation typically gives the value of d_max_ closest to what was measured by the Razor chamber.

**Table 2:** Dose measurements of d_max_ for a 1 × 1 and 3 × 3 cm^2^ square field

Field size (cm^2^)	Detector	Energy (MV)	*D_max_* (mm)
1 × 1	IBA razor diode	6X	11.65
	IBA razor chamber	6X	11.5
	PTW microDiamond (edge‐on)	6X	11.55
	PTW microDiamond (face‐on)	6X	12.2
1 × 1	IBA razor diode	6FFF	12.6
	IBA razor chamber	6FFF	10.45
	PTW microDiamond (edge‐on)	6FFF	10.9
	PTW microDiamond (face‐on)	6FFF	11.9
3 × 3	IBA razor diode	6X	13.3
	IBA razor chamber	6X	15.4
	PTW microDiamond (edge‐on)	6X	15.5
	PTW microDiamond (face‐on)	6X	15.2
3 × 3	IBA razor diode	6FFF	12.9
	IBA razor chamber	6FFF	13.2
	PTW microDiamond (edge‐on)	6FFF	12.4
	PTW microDiamond (face‐on)	6FFF	13.9

### 3.B Lateral beam profiles

**Fig. 6:**
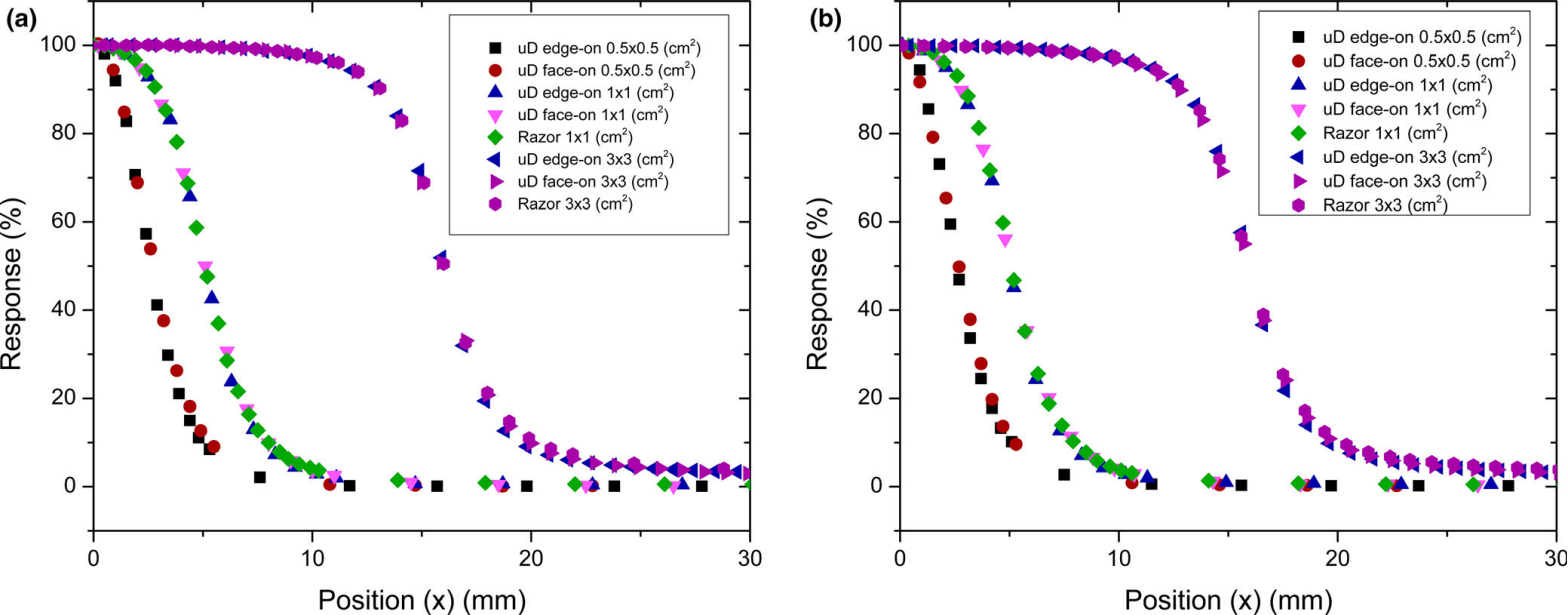
Cross‐plane profile measurements of 0.5 × 0.5, 1 × 1, and 3 × 3 cm^2^ jaw defined field Performed at 100mm depth in an IBA blue water two phantom on Varian TrueBeam with PTW microDiamond in edge‐on and face‐on orientations and Razor Diode for 6X (a) and 6FFF (b)

Jaw defined field profiles for sizes 0.5 × 0.5, 1 × 1, and 3 × 3 cm^2^ at 10 cm depth are presented in Fig. 6 for both 6X and 6MV FFF. The full width half maximum (FWHM) and average penumbra width for in‐plane and cross‐plane measurements for 0.5 × 0.5, 1 × 1, and 3 × 3 cm^2^ field are presented in Table 3. The penumbra is considered the distance between the positions of 80% and 20% of the normalized response. For the 0.5 × 0.5 cm^2^ field size, the FWHM measurements are between 1.1 and 4.4% narrower when measured with the microDiamond in edge‐on mode compared to face‐on. Similarly, penumbra widths are between 5.6 and 8.3% narrower when measured in edge‐on orientation. The face‐on orientation measures a larger or equal penumbra for all field sizes compared to the edge‐on orientation and is also larger or equal to measurements taken with the Razor diode from 1 × 1 cm^2^. The thinned sensitive volume of the edge‐on orientation produces a reduced volume averaging effect^31^ and is thus more appropriate for this measurement.

**Table 3:** FWHM and penumbra width for in‐plane and cross‐plane profile scans of a 0.5 × 0.5, 1 × 1, and 3 × 3 cm^2^ jaw defined field at 10 cm depth. Values to one decimal place

Field size	Detector	Energy (MV)	FWHM (mm)		Average penumbra (mm)	
			In‐planeCross‐plane	Cross‐plane	In‐planeCross‐plane	Cross‐plane
0.5 × 0.5 cm^2^	microDiamond (edge)	6X	5.4	5.2	2.3	2.4
	microDiamond (face)	6X	5.5	5.4	2.4	2.6
	microDiamond (edge)	6FFF	5.3	5.2	2.2	2.4
	microDiamond (face)	6FFF	5.3	5.4	2.3	2.6
1 × 1 cm^2^	IBA Razor diode	6X	10.5	10.2	2.8	3.1
	microDiamond (edge)	6X	10.4	10.2	2.9	3.0
	microDiamond (face)	6X	10.5	10.2	3.1	3.3
	IBA Razor diode	6FFF	10.4	10.5	2.6	3.0
	microDiamond (edge)	6FFF	10.3	9.9	2.8	3.1
	microDiamond (face)	6FFF	10.4	10.2	3.0	3.3
3 × 3 cm^2^	IBA Razor diode	6X	32.7	32.1	3.5	3.9
	microDiamond (edge)	6X	32.6	32.0	3.4	3.7
	microDiamond (face)	6X	32.6	32.0	3.6	3.9
	IBA Razor diode	6FFF	32.6	32.0	3.4	4.0
	microDiamond (edge)	6FFF	32.6	31.9	3.3	3.7
	microDiamond (face)	6FFF	32.5	31.9	3.5	4.0

### 3.C Output factor

**Fig. 7:**
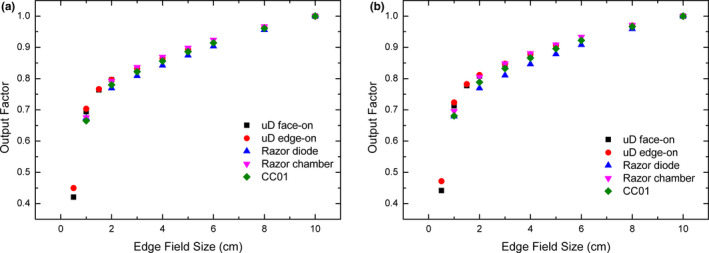
Raw OF measurements of MLC defined field performed on Varian TrueBeam with PTW microDiamond face‐on (Black square), microDiamond edge‐on (red circle), Razor diode (Blue triangle), Razor chamber (pink triangle), and CC01 (green diamond). OF measurements are presented for 6X (a) and 6FFF (b)

**Fig. 8:**
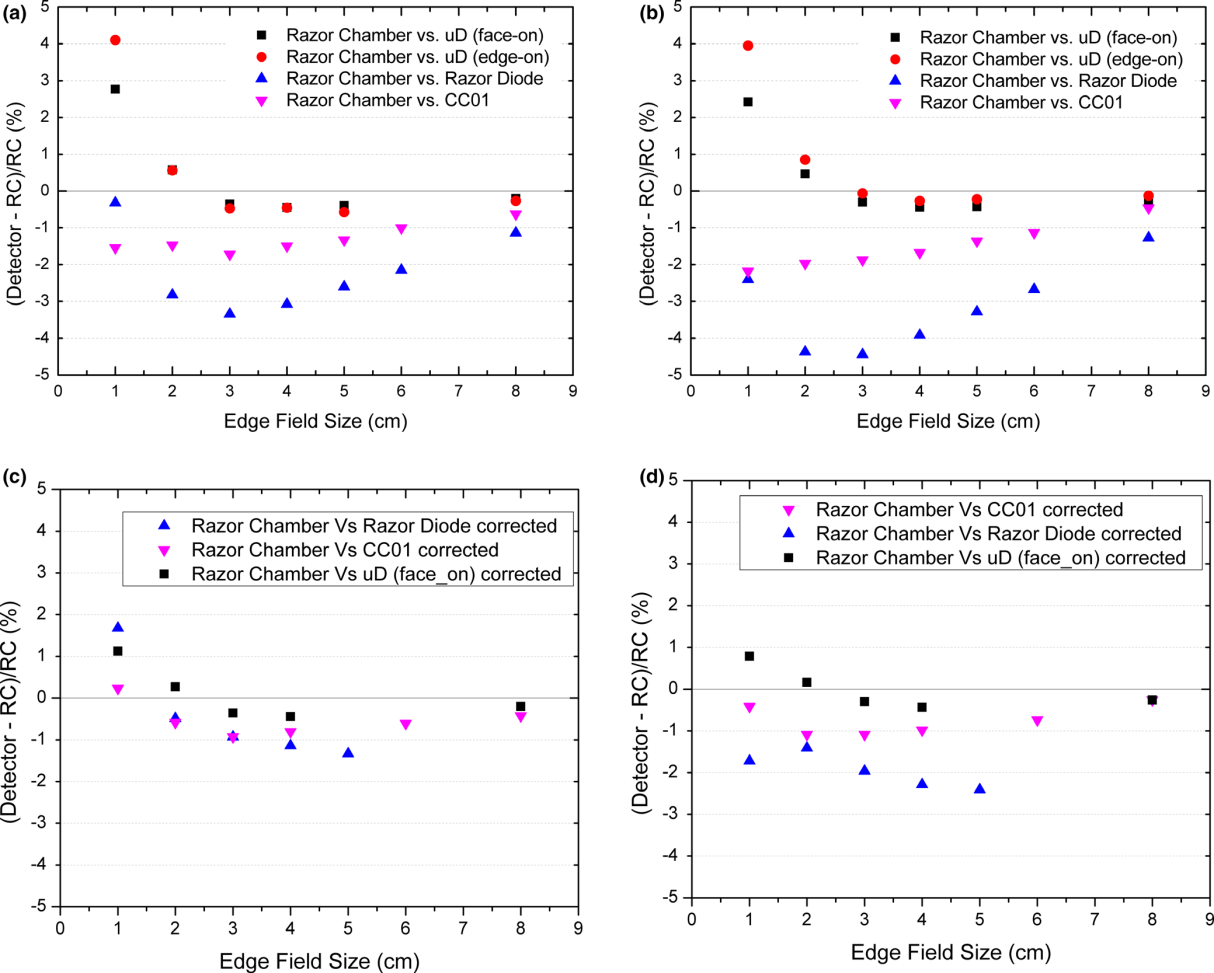
Percentage difference of raw OF for the PTW microDiamond in face‐on (black square), edge‐on (red circle), Razor diode (blue triangle), and CC01 (pink triangle) as compared to the IBA Razor chamber for 6X (a) and 6FFF (b). Percentage difference with correction factors applied 6X (c) and 6FFF d)

The photon output factor (OF) is shown in Fig. 7 for square field sizes from 0.5 × 0.5 cm^2^ up to 8 × 8 cm^2^ and are normalized relative to 10 × 10 cm^2^ field. Percentage difference graphs of the OF are shown in Fig. 8 reporting that the microDiamond in both orientations agrees within 1% down to the 2 × 2 cm^2^ field size for both energies. The OF measured with the PTW microDiamond (edge‐on) for the 1 × 1 cm^2^ field recorded an overresponse of 4.1% for 6X and 3.9% for 6FFF compared to the Razor chamber. The OF over response measured with the PTW microDiamond (face‐on) was 2.8% for 6X and 2.4% for 6FFF. The overresponse of the microDiamond at the 1 × 1 cm^2^ field size has been previously observed^2^, ^23^ and is related to three known effects — volume averaging, density perturbation, and radiation induced charge imbalance. They become prevalent only at small field sizes <2 cm in effective length. The density perturbation and radiation‐induced charge imbalance cause an overresponse where volume averaging causes an under response. Therefore the dominant effects are the density perturbation and radiation‐induced charge imbalance. At the 1 × 1 cm^2^ field size, the microDiamond in edge‐on orientation overresponds in respect to the face‐on orientation. This is due to a larger volume averaging effect in face‐on orientation compared with edge‐on, resulting in a lower OF. The difference between the two orientations increases further up to a difference in output factor of 4% for the smallest field size of 0.5 × 0.5 cm^2^, where the field is now smaller than the microDiamond detector volume when in face‐on orientation. Each point presented is an average of multiple measurements with a standard deviation of <0.2%.

The IAEA TRS‐483 CoP provides correction factors for the microDiamond and CC01 and these have been applied, where the data were available in Fig. 8. The data presented from a study by Poppinga et al.^6^ suggest that no significant correction factor is required to be applied to the Razor chamber until the field size is smaller than 1 cm^2^ and is therefore used as the reference chamber in Fig. 8. Correction factors from Casar et al.^32^ for a TrueBeam linac have been applied for the Razor diode in Fig. 8. For the 6X beam this brings all data points into agreement with the Razor chamber within 2%. For the 6FFF beam the correction factors bring the agreement between the Razor chamber and microDiamond within 1% and the CC01 and Razor diode within 2% and 3% respectively for all available points.

### 3.D Angular dependence

**Fig. 9:**
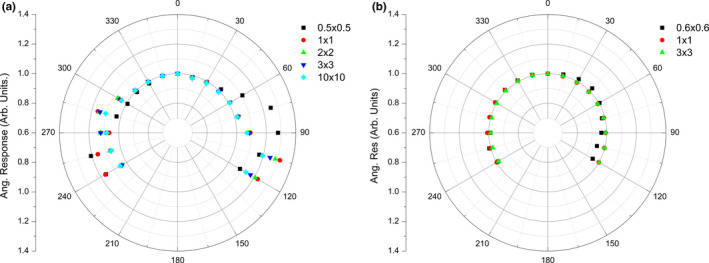
Angular dependence measurements as a function of gantry angle for a range of jaw defined fields performed with PTW microDiamond in the (a) face‐on phantom and (b) edge‐on phantom

Figures 9(a) and 9(b) depict the results of the angular dependence study of the PTW microDiamond detector within the cylindrical face‐on and edge‐on phantoms respectively (see Fig. 2). For the face‐on phantom [Fig. 9(a)] at 0° the microDiamond is in edge‐on orientation and at 270° it is face‐on to the beam. The largest deviations (up to ≈31%) for all field sizes were between angles 70° and 120°, likely due to a combination of the nonsymmetric nature of the packaging and the stem effect. The microDiamond is in edge‐on orientation for all gantry angles in the edge‐on phantom (Fig. 2) where no significant angular dependence is observed through the entire 240 range.

The variation in the response for face on‐orientation is reported in Table 4 with the data between angles 70° and 120° omitted. For this subset of angles, the microDiamond in face‐on orientation still has a significant angular dependence with the range of response as large as 12% at the 10 × 10 cm^2^ field. In face‐on orientation, the microDiamond is therefore highly angular dependent and would be inappropriate for measurements that involved multidirectional beam geometries.

**Table 4:** Face‐on angular dependence of microDiamond for 6X square fields upon a Varian Clinac IX. Angles include range from 240° to 60° normalized to 0° measurement

240° to 60°	0.5 × 0.5 cm^2^	1 × 1 cm^2^	2 × 2 cm^`^	3 × 3 cm^2^	10 × 10 cm^2^
Maximum response	1.21	1.16	1.15	1.14	1.10
Minimum response	0.99	0.98	0.98	0.98	0.98
Range	0.22	0.18	0.17	0.16	0.12

In the edge‐on phantom, the range in angular response through 240° reported in Table 5 shows only a 2% variation for the 2 × 2 cm^2^ and 3 × 3 cm^2^ fields. This increases up to 28% for a 0.6 × 0.6 cm^2^ field and is likely to be at least partially related to the introduction of jaw sag and/or a slight error in the vertical alignment of the detector which is only prevalent at very small fields. No fluence monitor chamber was used to correct for variations in machine output so this may also account for small variations. The main advantage of using the microDiamond in edge‐on mode is therefore an almost angular independent response even for small fields of 2 × 2 cm^2^. In Fig. 9(a) it is observed that smallest deviation in angular response is around the 0° position where the orientation has transitioned to edge‐on. In edge‐on mode, the microDiamond is therefore also insensitive to detector tilt.

**Table 5:** Edge‐on angular dependence of microDiamond for ± 120° around 0°. The PTW microDiamond is continuously in edge‐on orientation for all gantry angles for 6X square field upon a Varian Clinac IX

0° ± 120°	0.6 × 0.6 cm^2^	1 × 1 cm^2^	2 × 2 cm^2^	3 × 3 cm^2^
Maximum response	1.01	1.03	1.01	1.00
Minimum response	0.72	0.94	0.99	0.98
Range	0.28	0.08	0.02	0.02

### 3.E Stem effect

The values of the stem effect for the microDiamond are presented in Table 6 with the largest observed stem effect of 1*.*00% for the 1 × 3 cm^2^ field size. By the 3 × 10 cm^2^ field size, the stem effect is negligible for both energies recording less than a 0.2% increase in OF when the beam runs parallel to the cable. The close agreement of the stem effect between the 1 × 10 cm^2^ and the 1 × 3 cm^2^ suggests that the increased signal is primarily coming from interactions around the high Z electrodes of the microDiamond and not the cable.

**Table 6:** Percentage increase in output factor for a range of rectangular fields positioned along the cable of the microDiamond relative to when positioned perpendicular to the cable

Field size (cm^2^)	Stem effect (%)	
	6X	6FFF
1 × 10	0.83	0.93
3 × 10	0.19	0.17
1 × 3	0.80	1.00

### 3.F TPR_20,10_

The measurements of the TPR_20,10_(*S*) are presented in Table 7.The microDiamond has less than a 1% discrepancy to the CC01 at all field sizes. The Razor diode had a 2.61% difference to the CC01 for the 0.5 × 0.5 cm^2^ field and a difference of <1% at all larger field sizes. The IAEA TRS‐483 CoP suggests the use of ionization chambers for beam quality measurements due to the small mismatch in stopping power ratios between air and water, but also considers that the size of the detector should not perturb the field. The data presented in Table 7 show that the TPR does vary with field size and that the collimation of small field with MLCs will impact on beam quality. The close agreement between the microDiamond and the CC01 identifies the potential for microDiamond in edge‐on mode to be used for small field beam quality measurements, where its enhanced spatial resolution could be advantageous for measurement of small MLC shaped beams.

**Table 7:** TPR20_20,10_ (*S*) measured with the CC01, microDiamond in edge‐on orientation and Razor Diode for a 6X beam on a Clinac XI for a range of square field sizes. The percentage difference between the microDiamond to CC01 and Razor diode to CC01 are also included

Square field size cm^n^	CC01	microDiamond	% diff to CC01	Razor diode	% diff to CC01
0.5 × 0.5	0.585	0.590	0.80	0.600	2.61
1 × 1	0.613	0.613	0.00	0.615	0.33
1.5 × 1.5	0.618	0.620	0.32	0.621	0.49
2 × 2	0.620	0.623	0.48	0.623	0.48
3 × 3	0.626	0.625	−0.16	0.627	0.16
4 × 4	0.629	0.629	0.00	0.632	0.48
5 × 5	0.633	0.635	0.32	0.637	0.63
6 × 6	0.639	0.640	0.16	0.643	0.63
8 × 8	0.650	0.650	0.00	0.653	0.46
10 × 10	0.661	0.660	−0.15	0.664	0.45

## 4 DISCUSSION

It is evident the microDiamond in edge‐on orientation is not always appropriate for small field dosimetry. Nevertheless it provides a distinct advantage for particular measurements. When performing profile measurements, the edge‐on orientation provides improved FWHM and penumbra data for small fields, measuring up to 8.3% narrower penumbras. While this measurement orientation is advised against in the IAEA TRS‐483 CoP, due to the potential for extra cameral effect, the stem effect was shown to result in <1% increase in response. The main advantage of the adoption of an edge‐on orientation is an angular independent response for field sizes of 2 × 2 cm^2^ or greater with maximum 2% variation observed. Therefore, only in edge‐on mode would the microDiamond be appropriate for QA of IMRT and VMAT. A limitation of this angular dependence study being a full 360° study of angular dependence was not possible. Additionally, both orientations overresponded for OF measurements at small field sizes. Correction factors for the microDiamond OF in face‐on orientation have been created^33^ but future work will be required to determine corrections for the edge‐on orientation of the device.

The angular independent response of the microDiamond in edge‐on mode would make it a viable candidate for end to end QA with new rotation‐based systems, such as for MRI linacs.^33^ The angular dependence in edge‐on orientation is, however, not expected to be the same within a magnetic field. Inside the MRI linac the secondary electrons produced in the surrounding material are affected by the Lorentz Force depending on the field orientation.^24^, ^25^ The isolation and quantification of density perturbation effects on orientation will also be investigated in relation to the impact of magnetic field in future work.

## 5 CONCLUSION

The first characterization of the PTW microDiamond in an edge‐on orientation has been undertaken, demonstrating its advantages for dosimetry in small fields. The microDiamond in an edge‐on orientation has an angular independent response down to a 2 × 2 cm^2^ field with a maximum deviation in angular response of 2%. For a 0.5 × 0.5 cm^2^ field when edge‐on, the microDiamond on average measures the FWHM and penumbra between 1.1–4.4% and 5.6–8.3% narrower respectively, compared to the recommended face‐on orientation. The stem effect introduced when using the detector in edge‐on orientation was at a maximum producing a 1% increase in response. Both orientations require correction factors when taking measurements of field sizes <2 × 2 cm^2^ due to an observed overresponse. Correction factors for the edge‐on orientation will be the focus of future work. This study has demonstrated the advantages and potential versatility of using the microDiamond in edge‐on orientation for applications outside small field OF measurement.

## CONFLICT OF INTEREST

No conflict of interest.
